# Known and unknown: Exosome secretion in tumor microenvironment needs more exploration

**DOI:** 10.1016/j.gendis.2023.101175

**Published:** 2023-11-23

**Authors:** Mengxiang Huang, Jie Ji, Xuebing Xu, Dandan Jin, Tong Wu, Renjie Lin, Yuxuan Huang, Jiawen Qian, Zhonghua Tan, Feng Jiang, Xiaogang Hu, Weisong Xu, Mingbing Xiao

**Affiliations:** a Department of Gastroenterology, Affiliated Hospital of Nantong University, Medical School of Nantong University, Nantong, Jiangsu 226001, China; b Clinical Medicine, Medical School of Nantong University, Nantong, Jiangsu 226001, China; c Department of Nuclear Medicine, Affiliated Hospital of Nantong University, Nantong, Jiangsu 226001, China; d Department of Respiratory Medicine, Rudong County People's Hospital, Nantong, Jiangsu 226400, China; e Department of Gastroenterology, Affiliated Nantong Rehabilitation Hospital of Nantong University, Nantong, Jiangsu 226001, China

**Keywords:** Exosome cargo, Exosome secretion, Intercellular communication, Tumor, Tumor microenvironment

## Abstract

Exosomes, extracellular vesicles originating from endosomes, were discovered in the late 1980s and their function in intercellular communication has since garnered considerable interest. Exosomes are lipid bilayer-coated vesicles that range in size from 30 to 150 nm and appear as sacs under the electron microscope. Exosome secretion is crucial for cell-to-cell contact in both normal physiology and the development and spread of tumors. Furthermore, cancer cells can secrete more exosomes than normal cells. Scientists believe that intercellular communication in the complex tissue environment of the human body is an important reason for cancer cell invasion and metastasis. For example, some particles containing regulatory molecules are secreted in the tumor microenvironment, including exosomes. Then the contents of exosomes can be released by donor cells into the environment and interact with recipient cells to promote the migration and invasion of tumor cells. Therefore, in this review, we summarized the biogenesis of exosome, as well as exosome cargo and related roles. More importantly, this review introduces and discusses the factors that have been reported to affect exosome secretion in tumors and highlights the important role of exosomes in tumors.

## Introduction

**Figure 1:**
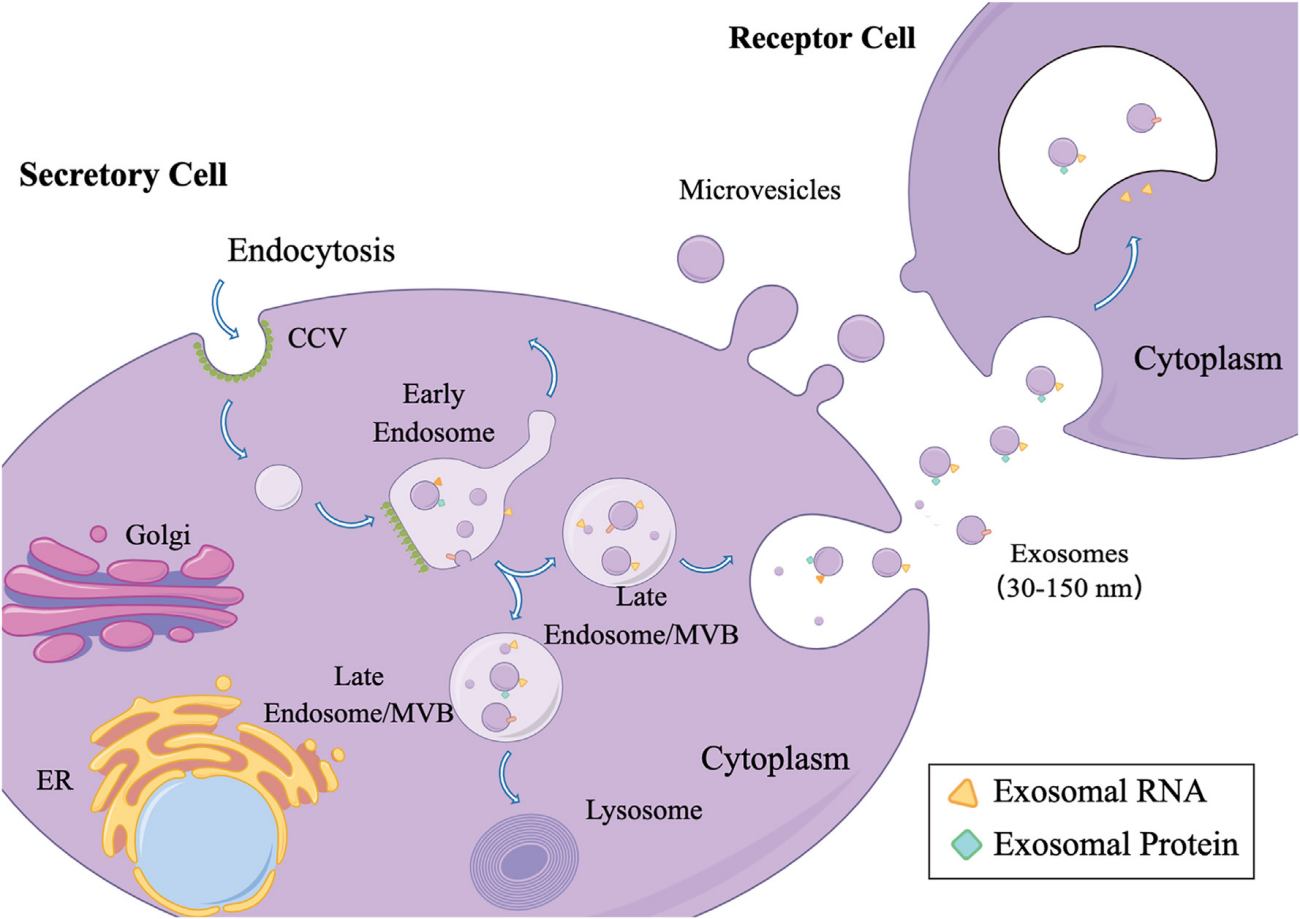
The generation pathway and outcome of exosomes. Early endosomes are created as a result of the plasma membrane of the cell invaginating, while late endosomes (multivesicular bodies, MVBs), which are multivesolar bodies carrying intraluminal vesicles, are created when the early endosomes continue to invaginate. MVBs are ultimately degraded by lysosomes or directly release intraluminal vesicles (known as exosomes).

Extracellular vesicles were originally identified in sheep reticulocytes in 1983 and subsequently named “exosomes” by Johnstone in 1987.^1^^,^^2^ Since then, it has aroused great interest among researchers and led to extensive studies. It is reported that extracellular vesicles are released *in vitro* through a variety of eukaryotic cell types, as well as being present in bodily fluids such as urine, blood, bile, synovial fluid, breast milk, and semen.^3^, ^4^, ^5^ Extracellular vesicles potentially act as biomarkers for liquid biopsies and therapeutic uses, and they now represent a novel paradigm for the transport of pathways for cell-to-cell communication.^6^ The biogenesis of exosomes originates from endosomes and interacts with related substances within the cell, ultimately producing the exosomes' final content, including nucleic acids, proteins, lipids, and metabolites.^7^^,^^8^ The four phases of exosome creation: cell membrane invagination, formation and maturation of multivesicular bodies (MVBs), and release of intraluminal vesicles are tightly regulated biological processes (Fig. 1).^9^

In addition to mediating normal physiological processes such as intercellular communication, immune response, and antigen presentation, exosomes also participate in pathological processes such as tumor invasion and migration.^10^^,^^11^ It is demonstrated that tumor cells produce excessive exosomes, which can have a certain impact on drug resistance, tumor development, and migration.^9^ Exosomes have been found to take part in various kinds of biological processes by transferring their payload to exosomes that are receptive to it.^12^

Exosome complexity in terms of composition, cargo molecules, and taxonomic proteins is represented by the multistep biological process known as exosome biogenesis, which is governed by a number of signaling channels. Different taxonomic mechanisms have been identified, though the precise exosome biogenesis mechanism is still unknown.^13^ Exosomes originate from endocytosis and develop from early endosomes and subsequently form late endosomes (MVBs), ultimately releasing exosomes.^14^^,^^15^ In this process, two mechanisms are involved: transport (endosomal sorting complex required for transport)-dependent and -independent processes, where transport-independent participates in the internal sorting of complexes.^16^ The exosomes finally have two results: one of that MVBs merge with the plasma membrane and release the intraluminal vesicles in it to the extracellular environment (now called exosomes), and the other is that MVBs merge with lysosomes and are degraded.^17^^,^^18^ Finally, exosomes can be “captured” by receptor cells, and their uptake occurs through different mechanisms of action, including membrane surface ligands binding to receptors, direct endocytosis into receptor cells, and direct fusion between membranes.^19^^,^^20^

The exosomes are mainly composed of three major components: protein, nucleic acid (DNA, miRNA, mRNA, lncRNA, circRNA, and tRNA), and lipids. The exosomes are rich in cholesterol and sphingomyelin and have a lipid bilayer structure.^21^ Meanwhile, exosomes contain high-level proteins such as transmembrane proteins (CD9, CD63, and CD81),^22^ heat shock proteins (Hsp 60, Hsp 70, and Hsp 90), and transport proteins (TSG-101 and Alix). These proteins remain on the surface of exosomes during secretion and are used as biomarkers for recognizing exosomes.^3^^,^^23^ Therefore, the factors affecting exosome secretion/release have been extensively studied. In this review, the process from the biogenesis to the release of exosomes is discussed, and some factors affecting the release of tumor exosomes, including exogenous stress, drugs, Rab family. These influencing factors may play a significant role in the clinical treatment of cancer. Therefore, we summarized and discussed about the recent advances in influencing exosome release in detail as follows.

## Exosomes' function in tumor disorders

Exosomes were once assumed to be “garbage cans” for cells' undesired substances, but it has now been shown that they serve multiple functions.^24^ Exosomes are natural nanovesicles, their size ranges from 30 to 150 nm, and because of their great biological permeability, excellent biocompatibility, and low immunogenicity, they have enticing advantages in cancer therapy.^25^, ^26^, ^27^

Exosomes are quickly becoming a potential tumor therapeutic option as medical research advances. Exosomes loaded with biological molecules or chemical drugs to the recipient's pathological site inhibit the metastasis of carcinogenic molecules and are expected to inhibit tumor progression.^28^, ^29^, ^30^ In particular, exosomes generated from tumor cells are considered to have preferential tumor delivery due to their homogenous characteristics.^31^ Scientists have conducted extensive research and reported on this one after another. In triple-negative breast cancer, it is expected to realize a new drug delivery system with CL4-modified exosomes, which can deliver lncRNA DARS-AS1 small interference to triple-negative breast cancer cells, and reduce the body's resistance to doxorubicin.^32^ Ginseng-derived exosome-like nanoparticles can penetrate the blood–brain barrier and target glioma cells, achieving significant therapeutic effects.^33^ In colorectal cancer, exosome lncRNA FAC1 derived from cancer-associated fibroblasts can alter its resistance to oxaliplatin.^34^ In gastric cancer, the application of Yiwei decoction in the treatment of spleen-derived exosomes can inhibit its progression and achieve a good therapeutic effect.^35^ Secondly, the immune regulation of tumor diseases also involves exosomes. For example, tumor-derived exosomes promote tumor growth by inhibiting T-cell activation through PD-L1.^36^ Exosomes influence tumor progression and metastasis and can induce effective pro-tumor and anti-tumor immune responses.^37^ Therefore, according to the function of immune cells, the use of immune cell-derived exosomes as anti-cancer agents to deliver drugs is expected to make a breakthrough in the treatment of tumors.^38^ SPION-modified neutrophil-derived exosomes achieve dual effects of targeting tumors and improving treatment by loading with doxorubicin.^39^ Tumor-associated macrophages affect tumor drug resistance and its progression, and exosomes derived from tumor-associated macrophages play an important role in the chemotherapy resistance of ovarian cancer.^40^^,^^41^ Natural killer cell-derived exosomes can effectively kill cancer cells through their inherent cytotoxicity.^42^ In addition, in cancer disease and cancer cells derived from outside the secreted body's unique characteristics, early cancer diagnosis and detection and high transfer of cancer cells can be achieved,^43^ heterogeneity can be assessed, cancer patients' response to treatment can be tracked, and testing in the treatment of the drug-resistant mechanism of potential biomarkers can help with accurate and personalized cancer therapy.^44^^,^^45^ Compared with traditional tissue biopsy, liquid biopsy can monitor tumor progression in real time.^46^ In recent years, research has shown that circulating tumor cells, circulating tumor DNA, and extracellular vesicles are the three core branches of liquid biopsy.^47^^,^^48^ Exosomes exist in body fluids such as blood, urine, and cerebrospinal fluid.^49^ More experiments have shown that extracellular vesicles are gradually becoming a promising indicator for cancer diagnosis, which has aroused widespread attention and research from more researchers.^50^ For example, the use of LSPR and AFM biosensors can detect increased levels of CD44, CD133, CD147, and MCT1 in malignant glioma-derived exosomes with greater accuracy.^51^^,^^52^ The combined detection of miR-128–3p and miR-33a-1p levels in serum exosomes or the levels of IGHV4-4 and IGLV-40 in plasma-derived exosomes using qRT-PCR is more sensitive for the diagnosis of non-small cell lung cancer.^53^^,^^54^ The piRNAs derived from serum exosomes have great potential in the diagnosis of hepatocellular carcinoma.^55^ Therefore, more research is needed to develop exosome-based liquid biopsy to provide a more convenient and affordable method for cancer diagnosis.

Meanwhile, exosomes derived from tumor cells can induce changes in other cells through epigenetics, thereby affecting tumor progression. Epigenetics refers to the changes in gene expression caused by non-sequence changes, including DNA methylation, histone modification, and non-coding RNA regulation (including miRNA).^56^^,^^57^ Based on researchers' findings, in breast cancer, exosomes secreted by breast cancer cells trigger p65 phosphorylation, and miR-183–5p is delivered to macrophages through exosomes to increase macrophage secretion, while exosome lncRNA C00657 induces the activation of M2 macrophages through m6A modification, thereby promoting tumor progression.^58^^,^^59^ Exosomes derived from esophageal squamous cell carcinoma can reduce the acetylation of lymphatic endothelial cells to promote lymph angiogenesis, but the specific regulatory mechanism remains to be studied.^60^ Based on the role of exosomes in cellular communication and epigenetics, researchers have been committed to studying their roles as biomarkers and drug carriers and their impact on tumor progression in recent years. However, achieving their future clinical application still requires the efforts of a large number of researchers.

**Figure 2:**
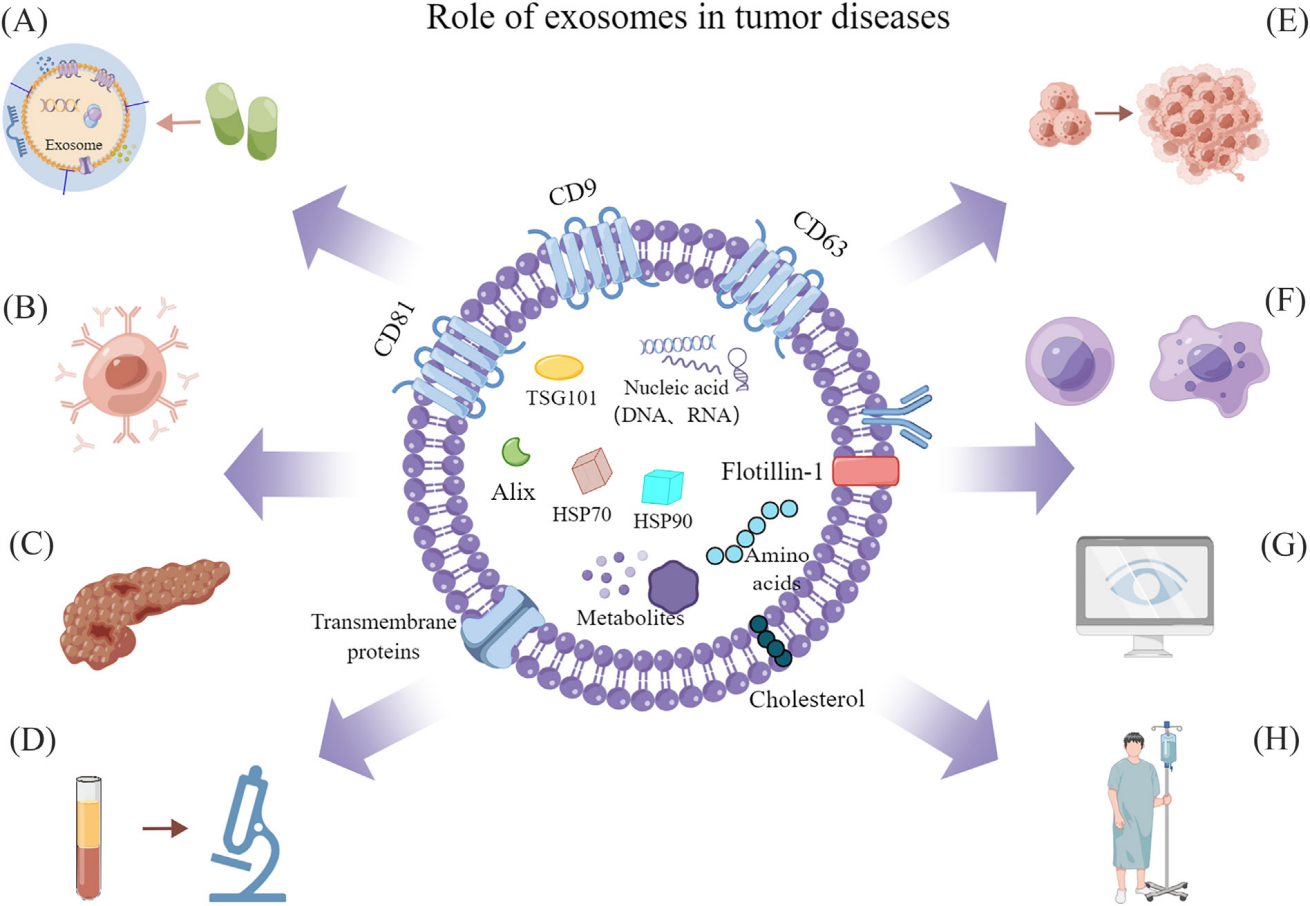
Role of exosomes in tumor diseases: **(A)** vehicles for drug delivery; **(B)** involved in immune regulation and anti-tumor immunity; **(C)** affect tumor progression and metastasis; **(D)** biomarkers for the diagnosis of early cancer; **(E)** detect highly metastatic cancer cells; **(F)** assess cancer heterogeneity; **(G)** track cancer patients' response to treatment; **(H)** reverse cancer chemotherapy resistance.

In short, exosomes can be selectively used for diagnosis, immunotherapy, RNA therapy, delivery of drugs as vectors, and reversal of chemoresistance in cancer.^61^^,^^62^ The underlying potential clinical role of exosomes in tumor diseases was disclosed (Fig. 2). Therefore, considering that the factors affecting exosome secretion may be used for clinical treatment in the future, this is an interesting area for further study and exploration. We believe that with the efforts of researchers, the clinical application of exosomes in tumor treatment is just around the corner.

## Exogenous stresses including hypoxia, pH change, and acute shear stress affect exosome secretion

Effects of hypoxia and hypoxic stress on exosome secretion hypoxia, defined as oxygen in tissues below 5–10 mmHg, is one of the most extensively researched aspects of tumor microenvironment (TME).^63^ The common internal environment of solid tumors is hypoxia, which is assumed to have a major role in promoting the occurrence and growth of tumors. In addition to inducing tumor cells' metabolic reprogramming to survive in the hypoxic TME, another major feature of hypoxia is to affect exosome secretion to promote tumor growth.^64^ Hypoxia-inducible factor (HIF) induces exosome secretion by boosting the expression and activation of cell surface protein receptors. HIF is not the only signaling molecule or pathway involved in the synthesis and exosome secretion under hypoxic conditions. Besides, signaling molecules and pathways include NF-kB, oxidative stress, PI3K/Akt/mTOR, and Rab-GTPase.^65^^,^^66^ Recent research has revealed that hypoxia leads to hypoxia-inducible factor-1α accumulation in head and neck squamous cell carcinoma and can inhibit the transcription level of V-ATPase in lysosomes, thus disrupting the acid balance of lysosomes. Reduced fusion with MVBs as a result of lysosomal dysfunction eventually results in increased exosome release.^67^ Another study showed that PTEN deficiency prevents TFEB-mediated lysosomal biogenesis, which enhances exosomal secretion and metastasis in cholangiocarcinoma.^68^ Hypoxia promotes the release of exosomes with glioma origins. Exosomes produced during hypoxia show increased autocrine and pro-migration activation of glioblastoma multiforme cells. Compared with normoxic exosomes, several miRNAs (miR-1246, miR-10b-5p, miR-182–5p, and miR-199a-3p) were up-regulated in hypoxic exosomes, leading to further deterioration of glioma.^69^ In hepatocellular carcinoma (HCC), hypoxia induces HCC cells to produce more exosomes. Studies have shown that hypoxic circumstances increase the expression levels of miR-1273f, which controls both Wnt/β-catenin signaling activation and directs replication in HCC to counteract exosome effects.^70^ High quantities of miR-210 are released by breast cancer cells in exosomes and hypoxic exosomes in a hypoxia-inducible factor-1α-dependent way. In addition, exosome numbers significantly increased when three distinct breast cancer cell lines were subjected to mild hypoxia (1% O_2_) and severe hypoxia (0.1% O_2_).^71^ Hypoxia promotes colorectal cancer (CRC) cells to release exosomes rich in miR-210–3p in the TME, which may contribute to the development of non-hypoxic CRC cells as preneoplastic phenotypes.^63^ Hypoxia has been demonstrated to greatly boost exosome secretion in ovarian cancer by down-regulating Rab 7, LAMP1/2, and NEU-1, up-regulating Rab 27a, and inducing more secretory lysosomal surface types.^72^ In pancreatic cancer, hypoxia induces pancreatic stellate cell activation and promotes exosome release.^73^ While pancreatic cancer cells can polarize macrophages by up-regulating a series of miRNAs when exposed to hypoxia and exosomes that are rich in miR-301a-3p.^69^ Exosomal secretion from different tumor cells is typically (though not always) enhanced by hypoxia through both direct (exosome biogenesis) and indirect (destruction of lysosomal acid microenvironments, influences of some regulatory factors, *etc*.) approaches. However, more thorough mechanisms are required.^74^

### Effect of pH change on exosome secretion

Exosome transportation or secretion in tumors may be significantly influenced by a few features of the TME. In addition to hypoxic conditions, the TME is acidic.^75^ Previous studies have found that exosome secretion is significantly increased at low pH, and the concentration of exosomal proteins and nucleic acids is significantly increased, while exosomal proteins, RNA, and other exosomal markers are not detected at high pH.^76^ Therefore, in pathological situations, the acidic TME may favor the fusion of exosomes and cells.

### Effect of acute shear stress on exosome secretion

Tumor cells that metastasize through the blood or lymphatic system must undergo acute shear stress.^77^ Acute shear stress is a significant component of the TME and is essential for the invasion and growth of the tumor. It is reported that shear stress causes autophagy in various kinds of cancer cells.^78^ Autophagy can regulate exosome secretion.^79^ For instance, acute shear stress increases the release of exosomes and autophagic elements from non-small cell lung cancer cells.^80^

**Figure 3:**
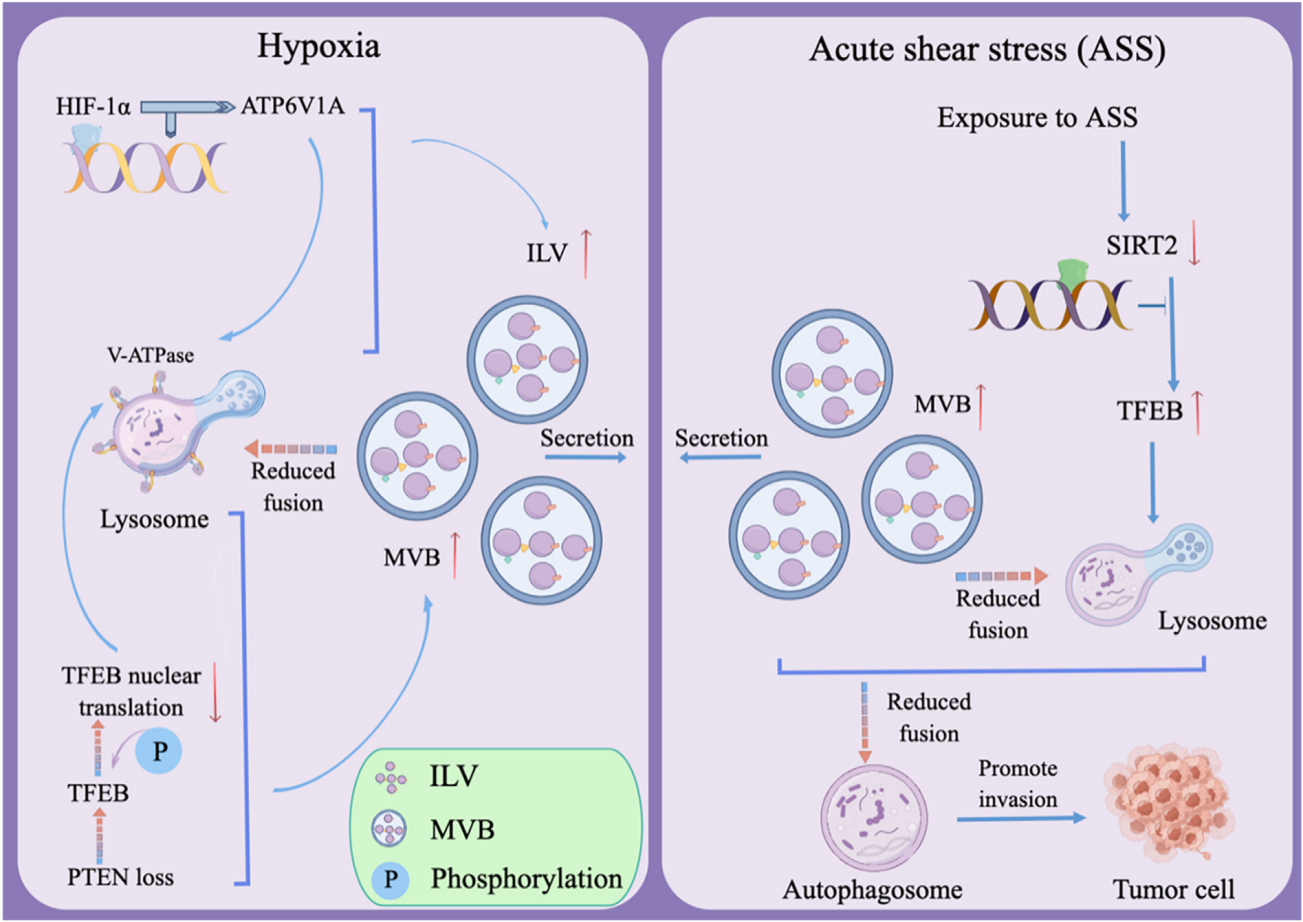
Hypoxia and acute shear stress can affect exosome secretion. Hypoxia can increase hypoxia-inducible factor-1α (HIF-1α) to inhibit the transcription of ATP6V1A in lysosomes, thereby destroying lysosomal function. Hypoxia leads to PTEN loss, followed by TFEB phosphorylation, which inhibits lysosomal biogenesis and ultimately promotes exosome release. Exposure to acute shear stress promotes exosome release by regulating the fusion of autophagosomes with lysosomes through TFEB. The increased release of exosomes accelerates tumor progression.

It is observed that in acidic or acute hypoxic, shear stress environments, the generation of cancer cells derived from outside the body secretion and secretion increases, and foreign body secretion-mediated cancer treatment is important, but its mechanism has not been well elucidated (Fig. 3). A more comprehensive understanding of the mechanism and in-depth studies were needed. It is challenging to improve the effect of hypoxic TME on exosome secretion and tumor progression in the future.

## Drug treatment affects exosome secretion

### Epalrestat

Epalrestat, an inhibitor of aldehyde-keto reductase family 1 member B (AKR1B1), is used to treat diabetic neuropathy, although its prospective utility and mechanism in the treatment of cancer are still unknown. However, we discovered that pancreatic cancer had higher AKR1B1 expression and linked favorably with metastasis. Exosome secretion is encouraged by up-regulated AKR1B1, which also speeds up pancreatic cancer cells' migration. Therefore, it may be possible to effectively stop the spread and growth of pancreatic cancer by pharmacologically targeting AKR1B1 with widely prescribed medications like epalrestat.^81^

### Amiloride

Amiloride, a pyrazine compound with potent potassium-preserving diuretic function, has been applied in clinical practice as a diuretic.^82^ Zhou et al^83^ demonstrated that tumor-derived exosome release was effectively inhibited by amiloride treatment and there was no appreciable impact on exosome release from normal tissues.

### Apatinib

A highly selective VEGFR2 inhibitor is apatinib, and the therapeutic effectiveness of apatinib in the treatment of advanced tumors has been shown in numerous clinical trials.^84^ Examples include Hodgkin's lymphoma,^85^ cervical cancer,^86^ metastatic gastric cancer,^87^ and advanced or recurrent biliary tract cancer.^88^ Zhao et al^89^ found that treatment with apatinib inhibits exosome secretion by modulating Lamp 2, Rab 11, Snap 23, and Vamp 2 to regulate MVB biogenesis, trafficking, and fusion.

### Matrine

Oxaliplatin has traditionally been the standard chemotherapy drug for CRC. Matrine is one of the active components in the extract of matrine, which has been found to inhibit tumors and enhance the anti-tumor activity of oxaliplatin against colon cancer. Studies have shown that matrine inhibits CRC by blocking exosomes released from cancer-associated fibroblasts.^90^

### Proton pump inhibitor

Proton pumps are crucial for tumor cells to survive in an acidic TME. Antiacids known as proton pump inhibitors work by inhibiting the proton pump in the cells lining the stomach wall, which helps to explain why gastric acid is secreted.^91^ Clinically, proton pump inhibitors are used in the treatment of peptic ulcers, gastroesophageal reflux disease, Droay syndrome, and upper gastrointestinal bleeding. The results showed that proton pump inhibitors at high doses inhibit the release of exosomes linked to tumor malignancy, which may be due to their effect in inhibiting the acidic TME.^92^ These include gastric cancer and HCC.^91^

### Simvastatin

Simvastatin is a hydroxymethylglutaryl CoA reductase inhibitor that not only inhibits the synthesis of endogenous cholesterol but also can prevent cardiovascular-related mortality and morbidity.^93^ Recent research has found that simvastatin can reduce exosome secretion in various cell types, which may be because simvastatin treatment significantly changes the close interaction between MVB-containing exosomes and the actin reticulum.^94^

### Metformin

The most commonly used diabetes medication in the world is metformin. Interestingly, metformin has been a potential candidate anti-tumor drug for the treatment of a variety of cancers in recent years, but its anti-tumor efficacy varies according to different cancers or subpopulations.^95^ According to certain research, metformin can suppress miRNA-21, miRNA-155, and miRNA-182 in U87MG human glioma cells, which results in the synthesis and secretion of exosomes being promoted.^96^

### Atorvastatin

The effectiveness of programmed death (PD-1/PD-L1) inhibition in the treatment of breast cancer can be enhanced by atorvastatin. Studies have shown that atorvastatin can reduce the level of PD-L1 in cancer cells, inhibit the secretion of exosomes, and reduce the content of PD-L1 in extracellular vesicles, thus improving the therapeutic effect.^97^

### Chemotherapy drugs

Chemotherapy is known to promote exosome release. For instance, cyclophosphamide was the first so-called “latent” broad-spectrum anti-tumor agent, effective in both leukemia and solid tumors. It has been found that cyclophosphamide can increase exosome secretion in sensitive cell lines without changing its anti-tumor properties in T-cell lymphoma for the past few years.^98^ Deoxy cytosine kinase activates the novel cytosine nucleoside derivative gemcitabine, the inactivation of which is catalyzed by cytosine nucleoside deaminase. It has been demonstrated that gemcitabine causes pancreatic cancer to secrete more exosomes.^99^ In addition, studies have shown that in human HCC cells, anti-tumor drugs (including carboplatin, irinotecan hydrochloride, paclitaxel, and etoposide) enhance the release of exosomes and generate more exosome-carrying heat shock proteins, which enhances the cytotoxic response's activation.^100^ In addition, researchers found that myeloma cells subjected to the anti-myeloma medications bortezomib, carfilzomib, or melphalan dramatically increased their exosome secretion.^101^

In summary, some drugs not only have good efficacy in the treatment of symptomatic diseases in clinical applications but also can affect the secretion of exosomes in tumor treatment to achieve therapeutic effects as found in medical experimental studies (Table 1). However, the specific mechanism by which some drugs affect the secretion of exosomes is still unclear, and more investigations are required for validation.

**Table 1:** Drugs affecting exosome secretion and mechanism of action.

Drug name	Drug action	For research on cancer	Mechanism	Reference
Epalrestat	Treatment of diabetic neuropathy	Pancreatic cancer	Affect the function of lysosomes	81
Amiloride	Treatment of edematous diseases	Cachexia of all kinds of tumors	By mediating anti-transporter proteins	83
Apatinib	Inhibition of tumor angiogenesis	Colorectal cancer, Hodgkin's lymphoma, cervical cancer, metastatic gastric cancer, recurrent or advanced biliary tract cancer	Affect the transportation and integration of multivesicular bodies	87
Matrine	Treating chronic hepatitis	Colorectal cancer	By regulating the expression level of CXR5	90
Proton pump inhibitor	Inhibition of gastric acid secretion	Liver cancer, stomach cancer, breast cancer	Affect the function of lysosomes	92
Simvastatin	Lipid regulation and anti-atherosclerosis	Colorectal cancer, liver cancer	The specific mechanism is unclear	93
Metformin	Reduce blood sugar levels	Human glioblastoma	The specific mechanism is unclear	96
Atorvastatin	Lipid regulation and anti-atherosclerosis	Breast cancer	By affecting exosome biogenesis	102
Chemotherapy drugs: cyclophosphamide, gemcitabine, carboplatin, irinotecan hydrochloride, paclitaxel, etoposide, bortezomib, carfilzomib, melphalan	Inhibit or kill tumor cells	Liver cancer, pancreatic cancer, lymphoma, myeloma, breast cancer, ovarian cancer, lung cancer, endometrial cancer, *etc*.	The specific mechanism is unclear	100

## Effects of some factors involved in exosome biogenesis on exosome secretion

### The Rab family influences exosome secretion

It is well known that members of the Rab family of small GTPases are involved in the intracellular transfer of vesicles.^102^ Rab GTPases regulate ordinated and dynamic intracellular membrane transport through the cytoskeletal pathway to maintain homeostasis. Rab GTPases are a crucial determinant in regulating vesicle motility in cancer.^103^ By facilitating the mobility of intracellular organelles and vesicles, Rab effectors, which are recruited to intracellular organelles by Rab GTPases, add to the specificity of membrane traffic.^104^ It has been found that Rab 5 and Rab 21 play roles in early endosomes, Rab 4, Rab 8, Rab 10, Rab11b, Rab 13, Rab 25, and Rab 35 play roles in circulating endosomes, and Rab2, Rab4, Rab 7, and Rab 9 participate in vesicle transport and exosome secretion. Rab 11, Rab 27, and Rab35 are involved in the merging of MVBs with the plasma membrane.^103^ Recent studies have found that Rab 37 and Rab 39 are new exosome regulators.^105^ Rab is converted from an inactive to an active state by guanine nucleotide exchange factor factors and GTpase-activating proteins.^106^ Rab in the active state localizes to specific vesicles and participates in exosome biogenesis.^107^ Rab5 is recruited and activated into the endosomes, and the activated Rab5 recruits more effectors, thereby promoting early endosome maturation.^108^^,^^109^ Mon1-Ccz1 could activate Rab7 on late endosomes, which in turn transported the late endosomal phase to lysosomes.^110^ Rab11 can promote MVB maturity by calcium ions and transfer them to the plasma membrane.^112^ Rab27 and Rab35 promote MVB fusion with the plasma membrane, but the specific mechanism of action remains unclear.^112^^,^^113^

### Rab members that directly affect exosome secretion

Rab5a is a crucial endocytosis regulator and participates in exosome release. Studies have shown that Rab5a affects the release of exosomes in human HCC cell lines. Rab5a may be a regulator of exosome secretion because stable silencing of Rab5a can reduce exosome secretion.^114^ The controlled secretion of lysosome-associated organelles by secretory granules, mast cells, platelets, and cytolytic T lymphocytes has previously been linked to Rab 27a and Rab27b. Recent studies have shown that Rab27a and Rab27b are crucial for targeting MVBs towards the periphery of cells and their docking on lipid membranes while promoting the secretion of exosomes.^112^ Sliencing of Rab27a and Rab27b inhibits exosome release. For example, Rab27a blockade in breast cancer cells reduces exosome secretion characterized by endocytic markers and leads to reduced primary tumor growth and apoptosis, as well as lung dissemination of metastatic cancer.^115^ In colorectal cancer stem cells, Rab27b has a unique role in controlling the exosomal pathway in colorectal cancer stem cells for TME regulation, and it has been demonstrated that targeting Rab27b is beneficial for anti-tumor therapy.^116^ Silencing the exocyst did not affect the interaction between MVBs and Rab11a while silencing Rab11a prevented the interaction between MVBs and the exocyst. From this, it can be seen that the exocyst serves as a junction in Rab11a-mediated transportation of MVBs to the plasma membrane.^117^ Inhibition of Rab11a expression in head and neck cancer can induce MVB accumulation and reduce the secretion of exosomes, thereby slowing down cancer development.^117^

### Regulating Rab and then affecting exosome secretion

The production, transportation, and metabolism of exosomes are crucial for human malignancies to develop and spread, and Rab GTPases are involved in all of these processes.^112^ Studies have shown that Rab 22a regulated by miR-193b influences exosome secretion in breast cancer cells.^118^ HOTAIR controls the level and localization of Rab 35 in HCC to promote the transportation of MVBs to the plasma membrane, thereby promoting exosome secretion.^66^ The mitochondrial protein TRX2 interacts with Rab35 in CRC and draws it to mitochondria. Rab35 is made to be ubiquitinated and degraded by TRX2, which impairs the secretion of exosomes and cell transmigration.^119^ Rab 31 promotes the release of exosomes from gastric cancer cells, thereby enhancing the invasion and migration ability of gastric cancer cells.^120^

**Figure 4:**
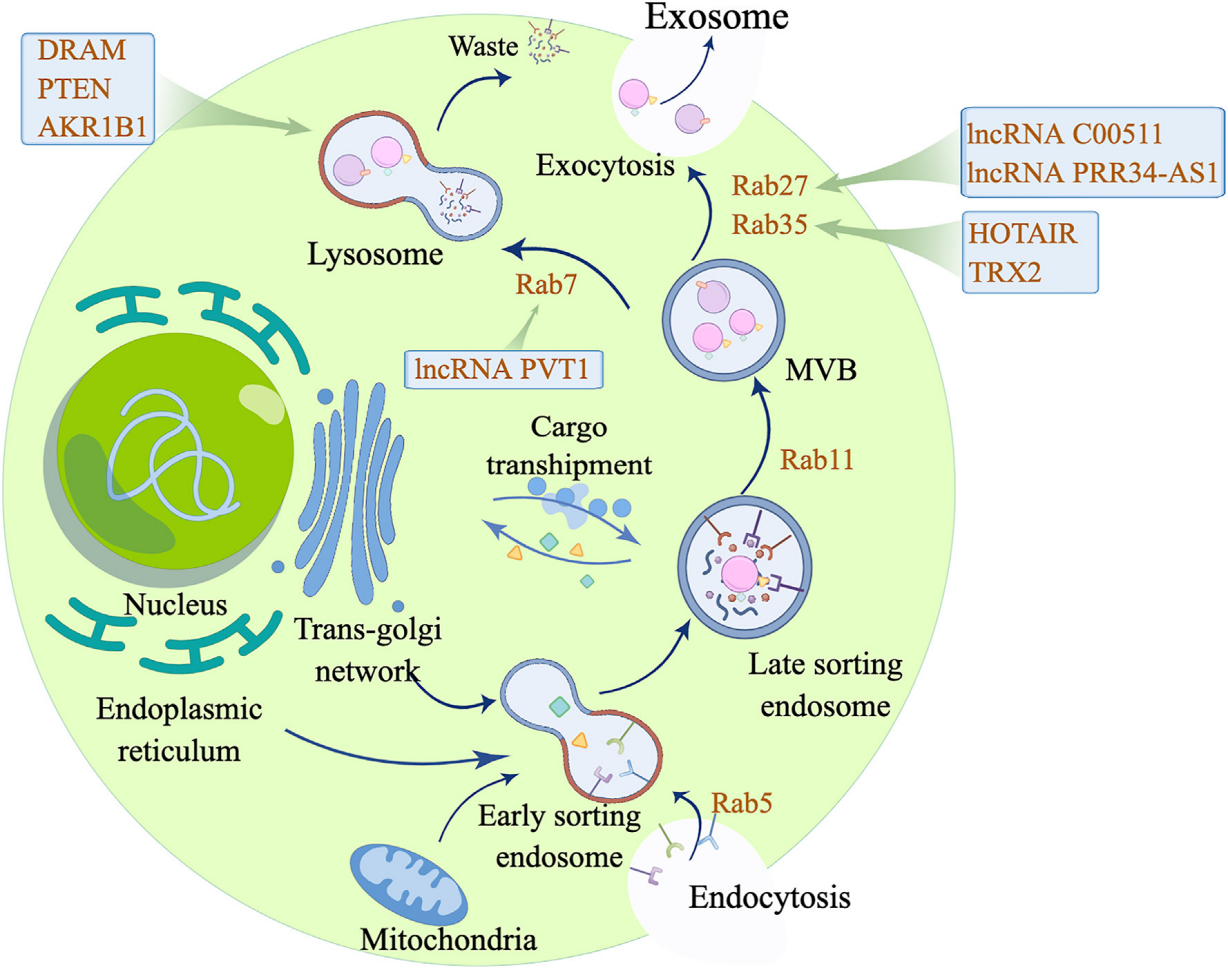
Influence of exosome biogenesis pathways. Rab 5 is a crucial endocytosis regulator involving in the formation of early endosomes (the earliest form of exosomes). Rab 11 promotes MVB maturation and guides MVB transport. Rab 7, which can be affected by lncRNA PVT1, mediates MVB transport to lysosomes for degradation. In addition, DRAM, PTEN, and AKR1B1 influence MVB degradation by regulating lysosomal function. Rab 27 and Rab 35 promote MVB fusion with the plasma membrane, which then releases intraluminal vesicles, known as exosomes. HOTAIR and TRX2 are essential molecules that regulate RAB35 levels. RAB27 is regulated by lncRNA C00511 and lncRNA PRR34-AS1. MVB, multivesicular body; PVT1, plasmacytoma variant translocation 1; DRAM, damage-regulated autophagy modulator; AKR1B1, aldo-keto reductase 1 member B1; HOTAIR, HOX transcript antisense RNA; TRX2, thioredoxin 2.

In short, Rab GTPases are recognized to be essential for exosome formation and to mediate exosome secretion (Fig. 4). However, the molecular mechanism by which Rab GTPases affect exosome secretion in tumor cells has not been better elaborated and further investigation is still needed.

### The effects of lipids and their constituent cholesterol on exosome secretion

Exosomes contain a significant amount of lipids, and numerous researches have demonstrated that these lipids are crucial to the biology of extracellular vesicles, which are rich in sphingolipids, cholesterol, and phosphatidylserine than donor cells.^121^ Lipid-induced damage-regulated autophagy modulator can interact with stomatin in hepatocytes, and damage-regulated autophagy modulator is a lysosomal protein that is crucial for exosome secretion. Studies have shown that impaired lysosomal function reduces the degradation of MVB-containing exosomal precursors by lysosomes, resulting in more MVB merging with the plasma membrane, which in turn increases exosome secretion.^122^ In particular, cholesterol is one of the components of lipids. Breast cancer pathogenesis and progression are linked by cholesterol and its metabolites. Breast cancer patients with elevated circulating total cholesterol have a worse prognosis. 27-hydroxycholesterol, a key component of cholesterol, has a multifaceted impact on cancer biology and could stimulate exosome secretion in breast cancer, thereby promoting breast cancer progression.^123^ In addition, cholesterol can induce lysosomal dysfunction to increase exosome secretion from hepatocytes, which in turn causes M1 polarization and inflammation brought on by macrophages in a way that is dependent on miR-122–5p.^124^

### Effect of adiponectin on exosome secretion

Only adipocytes secrete adiponectin, an unusual substance that is present in large quantities in the peripheral circulation and exists as trimers, hexamers, and high molecular weight mulers.^125^ A glycosyl linderia adenosine-anchored cadherin called T-cadherin that binds to the cell surface, accumulates in MVBs, and increases exosome formation and secretion is what adiponectin does to cells that express it.^126^ Adiponectin has numerous diverse effects, including direct impacts on tumor cell metabolism, the TME, and tumor cell signaling. According to epidemiological studies, breast cancer exhibits more aggressive features, including higher histological grade, angiogenesis, and metastasis, among women with low levels of circulating adiponectin.^127^ However, the molecular basis of adiponectin in breast cancer remains to be understood and elucidated.

### The effect of lncRNAs on exosome secretion

LncRNAs are non-coding RNAs, which are essential for cancer progression and can regulate the growth and spread of the tumor.^128^ For instance, lncRNA plasmacytoma variant translocation 1 promotes MVB docking by changing Rab 7 expression and localization in pancreatic cancer, thereby promoting exosome secretion in pancreatic cancer cells.^129^ But more research needs to be done on the function of the lncRNA plasmacytoma translocation 1 in pancreatic cancer exosomes. In HCC, lncRNA nuclear paraspeckle assembly transcript 1 can change exosome secretion produced by HCC cells and promote cell invasion and growth through down-regulating miR-634, miR-628, and miR-3960.^130^ The lncRNA PRR34 antisense RNA 1 (PRR34-AS1) is elevated in HCC cells. Silencing PRR34-AS1 can prevent HCC cells from proliferation, migration, invasion, and EMT phenotype. However, PRR34-AS1 up-regulation accelerates the malignant phenotype of THLE-3 cells. In addition, PRR34-AS1 promotes exosome secretion in HCC cells by increasing the expression of Rab 27a and promotes the malignant phenotype of THLE-3 cells.^131^ Meanwhile, researchers found that lncRNA C00511 induced the invader development by inducing MVB docking and exosome release. Exosome release from HCC cells has been shown to be induced by lncRNA C00511.^132^ In addition, HULC (highly up-regulated in liver cancer) is a lncRNA in HCC, which has been found to be an important regulator of HCC progression. A bioinformatics investigation revealed that the miR-372–3p/Rab11a axis was the mechanism through which HULC mediated exosome secretion from HCC cells. Another study indicated that HULC could stimulate the release of exosomes.^133^

## Other factors that affect exosome secretion

For example, syntaxin 2 is a key member of the synaptic fusion protein family, which is implicated in vesicle genesis and HCC metastasis. Recently, it was found to promote CRC metastasis, which is closely related to the increase in exosome secretion by syntaxin 2 in CRC cells.^134^ The elevation of intracellular calcium is essential for the growth and spread of tumors. Especially in breast cancer and lung cancer, Munc 13-4 is an important regulator of Ca^2+^-stimulated exosome release in metastatic cells, and the acute increase in Ca^2+^ in cancer cells stimulates a significant amount of exosome release.^111^ In response to stress signals, the p53 protein controls the transcription of multiple genes. In lung cancer, p53 up-regulates the transcription of TSAP6, a multichannel transmembrane protein, to increase exosome release after DNA damage.^135^ In HCC, DDX3 can regulate the secretion of exosomes from HCC cells to exert its tumor-suppressive effect.^136^ Some of the factors affecting the secretion of exosomes in tumor diseases have been discussed above, and there are other factors influencing the secretion of exosomes. For example, alcohol consumption can cause oxidative stress and promote exosome secretion. It is demonstrated that exposure of human HCC to alcohol induces increased exosome release by regulating Rab proteins, vesicle-associated membrane proteins, and protruding fusion proteins directly or partially through miR-192.^137^ In multiple tumors, Golgi phosphoprotein 3 interacts with cytoskeleton-associated protein 4 and reduces the amount of cytoskeleton-associated protein 4 localized in the plasma membrane. Furthermore, the Golgi phosphoprotein 3/cytoskeleton-associated protein 4 axis promotes exosome secretion.^138^ A high-fat diet mediates AMPKα1 inhibition and promotes nonalcoholic fatty liver disease by increasing the number of exosomes in CD63-containing adipocytes.^139^ Even exercise, a basic intervention, helps reduce the risk of many diseases, such as cancer and may be related to the promotion of exosome secretion by exercise.^140^

## Conclusion

**Figure 5:**
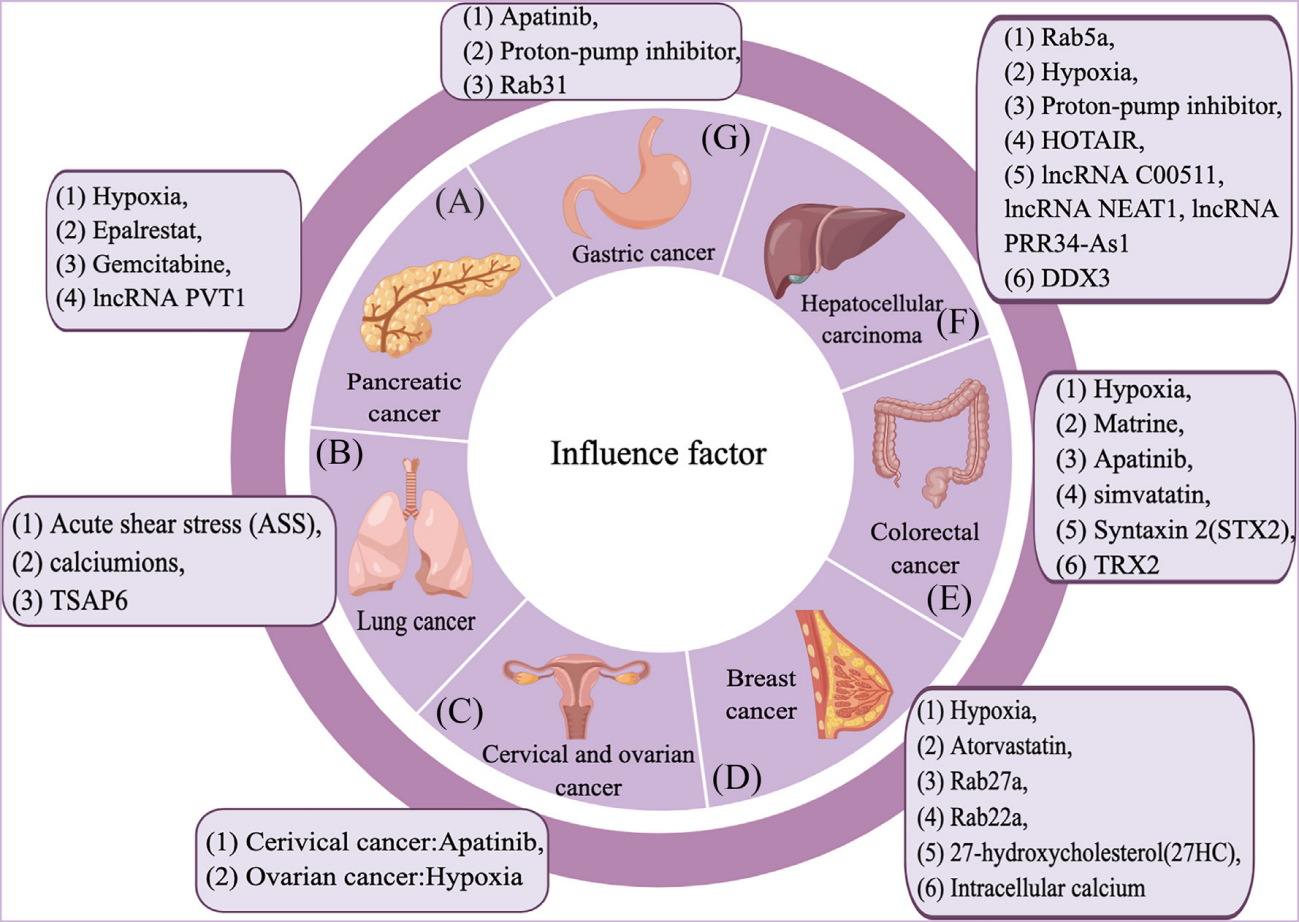
Factors affecting the release of exosomes from various organs under tumor conditions.

The factors that influence the secretion of exosomes have been investigated more and more in recent years. Thus far, we have summarized the above factors affecting exosome release and revealed their roles in exosome secretion in tumor diseases (Fig. 5). Among them, the TME is a significant site for the biogenesis and secretion of exosomes and could be affected by environmental changes. With the increase in tumor incidence, more drugs have been applied to clinical treatment, and some of them can achieve better therapeutic effects by affecting the release of exosomes. In addition, factors related to exosome biogenesis, such as the Rab family, lipids, and lncRNAs, can directly or indirectly affect exosome secretion in tumor diseases. Moreover, alcohol drinking and a high-fat diet could impact exosome secretion to some extent. However, the molecular mechanism regulating the secretion of exosomes derived from other tumor cells (such as immune cells) is not described. In the past few decades, scientists have carried out in-depth research in the field of cancer-based on the molecular mechanism of regulating the secretion of exosomes, and exosomes are of great value in tumor treatment and diagnosis. Therefore, the potential relationship between exosomes and tumors still needs deeper and more detailed research. Although the current research is still in the preclinical stage, it is believed that exosomes will show their value in clinical applications in the near future.

## Author contributions

**Mengxiang Huang and Mingbing Xiao:** Conceptualization. **Mingbing Xiao, Weisong Xu and Xiaogang Hu:** Supervision and Project administration. **Mengxiang Huang:** Writing-Original Draft. **Mengxiang Huang, Jie Ji and Xuebing Xu:** Visualization. **Jie Ji, Xuebing Xu, Dandan Jin, and Yuxuan Huang:** Investigation and Writing-Reviewing and Editing. **Tong Wu, Renjie Lin, Jiawen Qian, Feng Jiang and Zhonghua Tan:** Writing-Reviewing and Editing.

All appropriate contributors are listed as authors and all authors have agreed to the manuscript's content and its submission to Genes and Diseases.

## Conflict of interests

The authors declare that they have no competing interests.

## Funding

This study was supported by grants from the 10.13039/501100001809National Natural Science Foundation of China (No. 82272624), the 10.13039/501100004608Natural Science Foundation of Jiangsu Province, China (No. BK20211105), the Plan of Jiangsu Provincial Medical Key Discipline (China) (No. ZDXK202240), the Social Development Foundation of Nantong City, Jiangsu, China (No. MS22022044, JC22022001, JCZ2022027), the Health Project of Nantong City, Jiangsu, China (No. MS2022056), the Postgraduate Research and Practice Innovation Program of Jiangsu Province, China (No. SJCX21_1452, KYCX21_3112, KYCX23_3419), Jiangsu Provincial Research Hospital (China) (YJXYY202204), the Jiangsu Administration of Traditional Chinese Medicine (China) (JD2022SZ07), and Teaching Research Project of Affiliated Hospital of Nantong University (China) (Tfj22002).
